# Complex Reconstruction of a Mangled Forearm Following Washing Machine Trauma

**DOI:** 10.7759/cureus.13673

**Published:** 2021-03-03

**Authors:** Matthew Wolfers, Feargal Geraghty, Lisandro Montorfano, Stephen J Bordes, Glenn Miller

**Affiliations:** 1 General Surgery, Cleveland Clinic Florida, Weston, USA; 2 Trauma Surgery, Kendall Regional Medical Center, Miami, USA; 3 Surgical Anatomy, Tulane University School of Medicine, New Orleans, USA

**Keywords:** hand, upper extremity, trauma, reconstruction, tendon transfer

## Abstract

Automatic washing machine injuries are more commonly associated with minor injuries in the pediatric population but may cause life and limb-threatening adult injuries in rare instances. This case describes a severe upper extremity injury after a schizophrenic patient placed her arm into a running machine. Herein, we describe the management, complex reconstruction, and repair of radial, ulnar, and metacarpal fractures in addition to transected tendons and vasculature. The patient had an excellent functional outcome with minor restrictions in motion and complete recovery of sensation.

## Introduction

Automatic washing machines have caused traumatic injuries since their production began in 1910 with the introduction of electric Wringer washing machines [1]. 12.8% of automatic washing machine injuries reported by the United States Consumer Product Safety Commission involved the placement of an upper or lower extremity into a running machine [2]. And, 51.9% involved one or more bone fractures [2]. Herein, we present a case of an adult female suffering from a mangled upper extremity after placing her arm into a running washing machine. The patient had excellent functional outcome following extensive surgical reconstruction. We describe the appropriate steps leading up to the assessment and repair of life and limb-threatening injuries in a rare situation.

## Case presentation

A 59-year-old woman with a history of schizophrenia was brought to the trauma bay by ambulance after injuring her right upper extremity by placing it into a running automatic washing machine. Extrication took approximately 20 minutes and paramedics placed a tourniquet proximal to the elbow to stop the bleeding.

On arrival at the trauma bay, the patient was alert, tachycardic, tachypneic, and hypotensive. Right-hand digits were mottled and cyanotic; and the orientation of the hand to the arm did not appear anatomic, suggesting a displaced fracture. She was sedated and intubated. Upon removal of the dressing, an open fracture of the distal radius was visible with active bleeding despite the tourniquet. The tourniquet was briefly taken down, but no pulsatile bleeding was evident. A radiograph of the right forearm showed a displaced comminuted radial fracture and markedly displaced oblique fracture of the distal ulna with fragmentation. She was transported to the operating room for repair.

**Figure 1:**
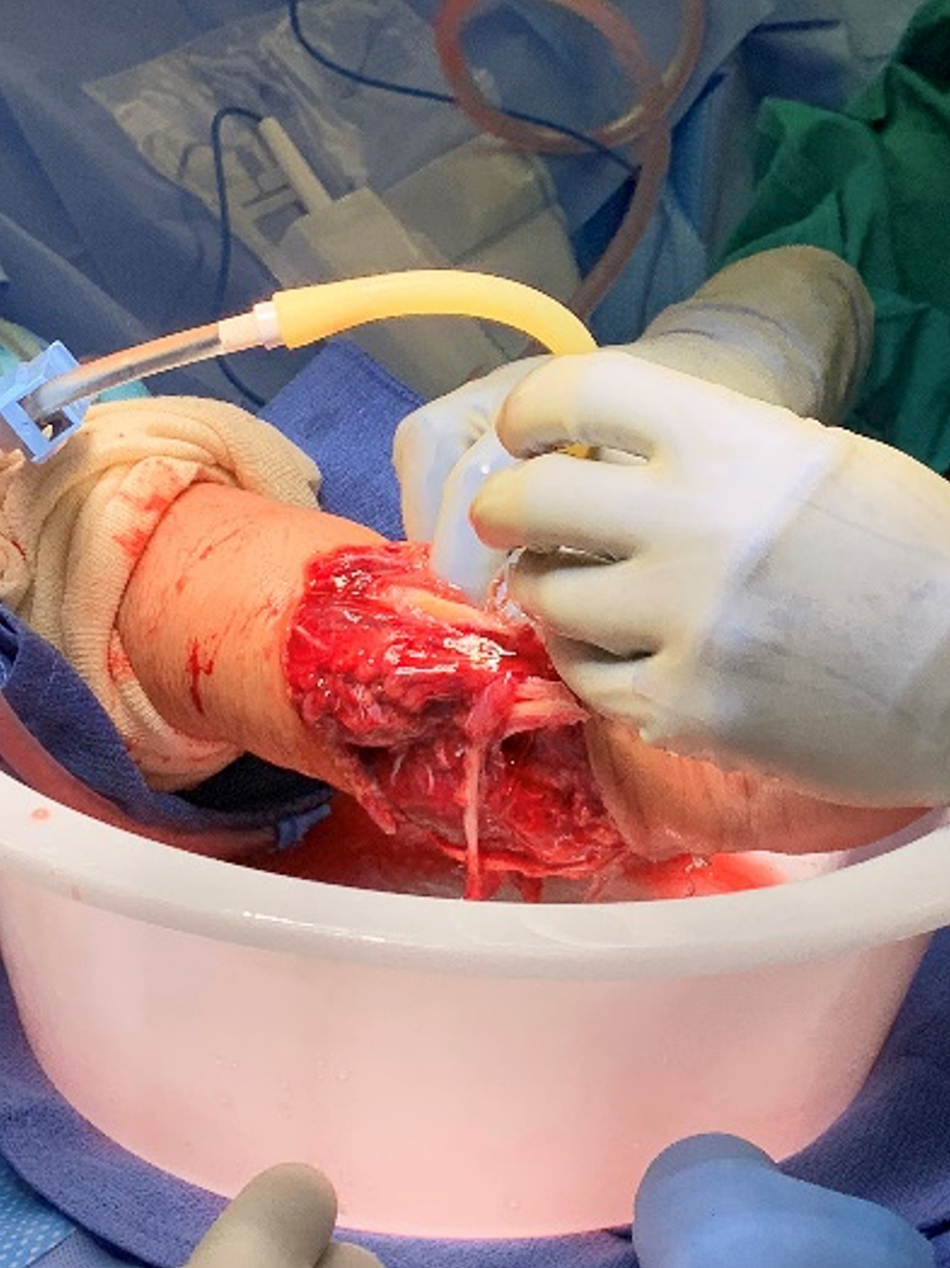
Exploration and irrigation of the right forearm

**Figure 2:**
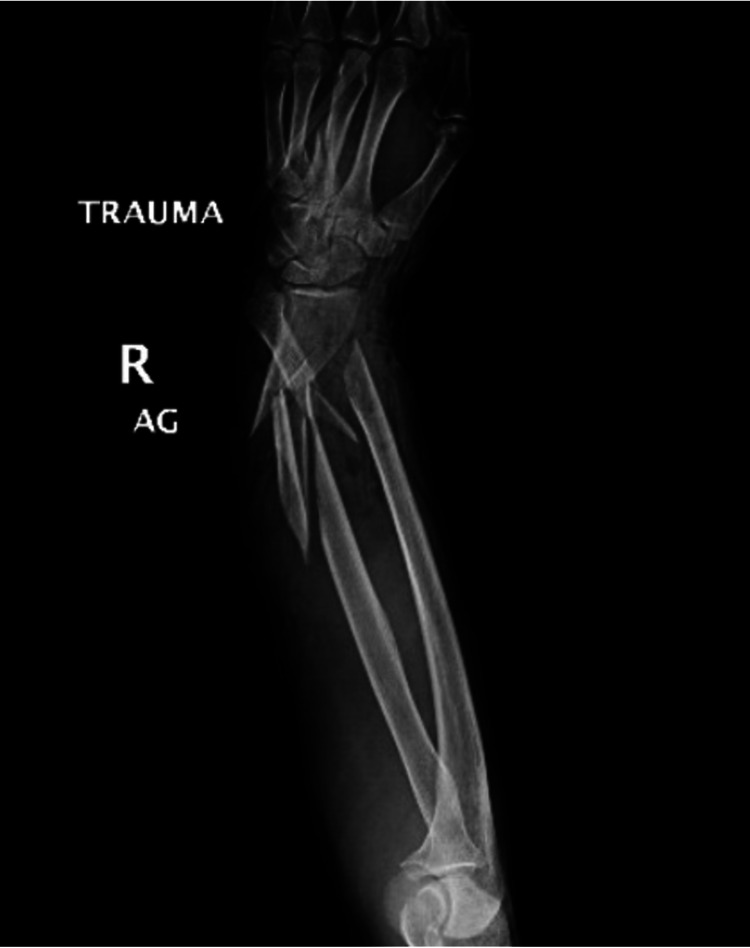
Radiograph of right upper extremity demonstrating ulna, radius, fourth metacarpal, and fifth metacarpal fractures R - Right AG - Silver

In the operating room, the wound was further explored (Figure 1), and additional radiographs were taken (Figure 2). No major vascular injuries were identified and mottling improved when the tourniquet was removed. She was found to have additional closed comminuted fractures of the right fourth and fifth metacarpals as well as disruptions of the extensor digitorum communis tendon, extensor digiti minimi, extensor pollicis longis, adductor pollicis longus, and flexor carpi ulnaris.

**Figure 3:**
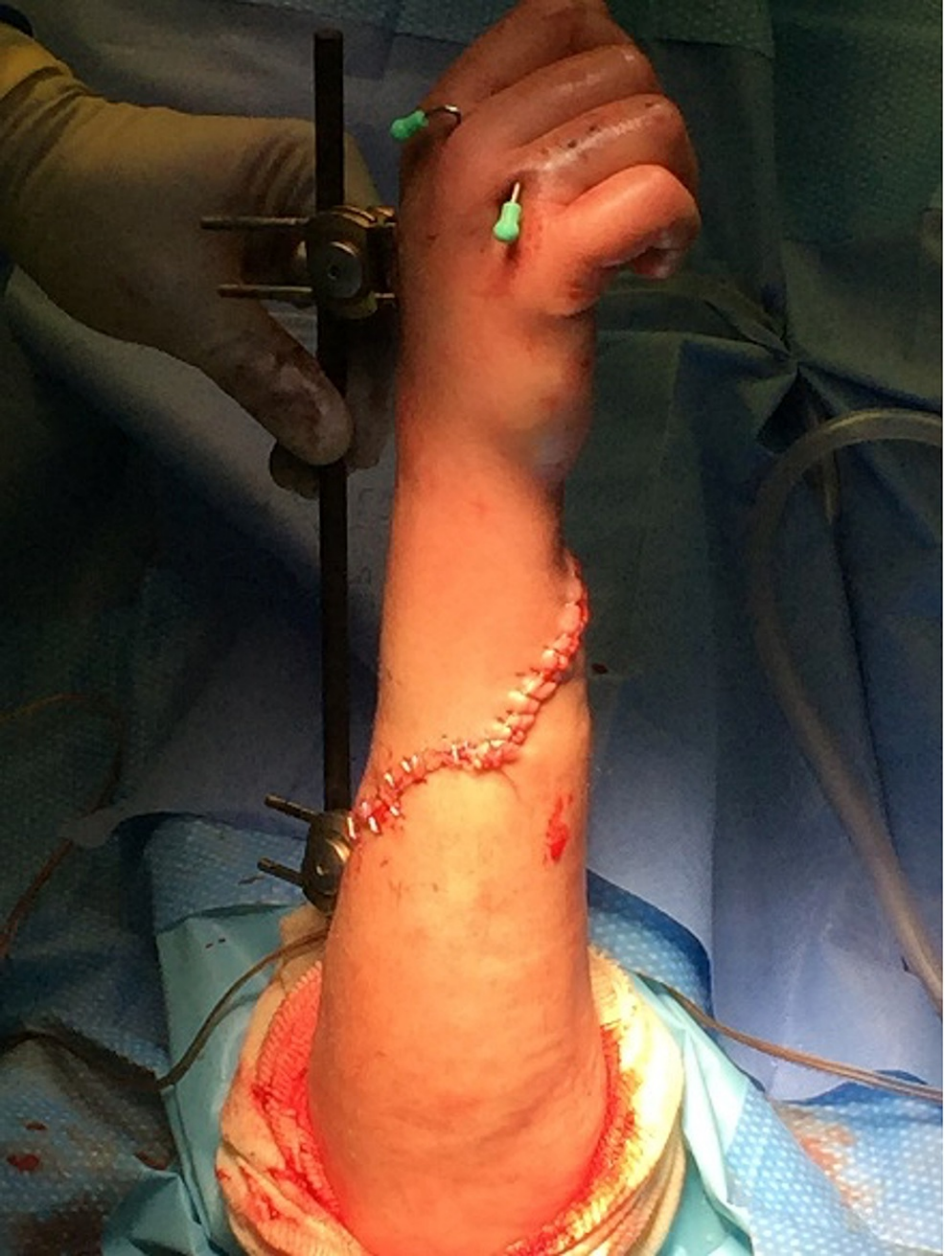
Right upper extremity after complex repair with external fixator and metacarpal pins

The wound was irrigated and debrided of devitalized tissue, and an external fixator was placed to reduce the radial and ulnar fractures. The metacarpal fractures were fixated with pins. Tendons were primarily repaired, except for the extensor digiti minimi tendon, which was transferred to the extensor digitorum communis tendon. The 23cm wound was then closed around the fixator screws with staples, and a splint was applied (Figure 3). She tolerated the procedure well and was transferred to the trauma intensive care unit for hourly vascular checks.

The patient’s post-operative course was uneventful. She initially had paresthesia of the right first and second digits, but sensation fully returned and all digits moved appropriately two days later. She was discharged on post-operative day 10 with a peripherally inserted central catheter for two weeks of antibiotics. The patient returned to the operating room two months later for corrective osteotomy of right ulnar and radial shaft malunion, open reduction and internal fixation of ulnar and radial shafts, reconstruction of the right radial shaft with bone autograft and demineralized bone matrix, and removal of implanted hardware from the right hand. At her nine-month follow-up, the patient had recovered well with a minor limited range of motion of the fingers, hand, and wrist for which she was working with specialized physical therapists.

## Discussion

Modern automatic washing machines have implemented numerous safety mechanisms to prevent injury during operation, but there are older machines in use, especially among lower socioeconomic populations [3]. Devastating injuries are uncommon but may require limb salvage techniques when they do occur.

When treating an injured limb, it is vital to treat life-threatening injuries first before addressing the limb, unless the limb is the source of catastrophic bleeding [4,5]. Bleeding is frequently controlled with pressure; but there are situations, such as this case, in which a tourniquet is appropriate [4]. Although tourniquets can cause nerve injury as well as limb ischemia, they have been shown to have a very low rate of complications when applied in the field prior to hospital admission [6,7]. After relative hemostasis, the wound must be assessed next to determine the extent of the injury.

Initially, the status of limb perfusion must be determined, and vascular injuries should either be repaired or temporized using a shunt if the vascular repair would hinder further reconstruction [4]. After the administration of antibiotics, devitalized tissue should be debrided in the operating room. Timing for debridement has been controversial, but multiple studies have shown that there is no difference in outcomes if done within 24 hours [4,7]. In this case, the decision was made to debride immediately due to mottled digits and bony instability.

Attention then turns to focus on any fractures. An external fixator should be considered when temporary stabilization of the limb is necessary [8]. We applied an external fixator for stabilization to allow for the repair of the extensive muscular and tendinous injuries. Bone shortening can be used for devitalized bone and improved approximation [8]. Bone grafts can be used for defects less than 6cm, and vascularized fibular bone or cement spacers with negative pressure dressings can be used for bone defects greater than 6cm depending on the level of contamination [8].

Tendon contracture begins as early as the initial debridement, so tendinous injuries should be addressed as soon as soft tissue coverage can be guaranteed [8]. Primary repair using nonabsorbable sutures can be performed if there is no tension; but a tendon graft, commonly using a side-to-side or weave technique, may be necessary to achieve a tension-free repair [9]. In this case, we were able to primarily repair multiple tendons; however, we resorted to tendon transfer for the extensor digiti minimi. This is useful for more extensive tendon injuries and allows for early active motion as the injured tendon is incorporated into a different functional muscle unit [8]. Tenodesis is a last resort if there are no other options for repair [8].

Although no gross nerve injuries were visualized in this case, nerve injuries must be addressed to restore function to the limb. Transections can be treated with autograft, most commonly using the sural nerve, allografts, or conduits for small gaps [8,10]. Timing of nerve repair remains controversial with some sources recommending repair within the first few days and others recommending a delay of up to three weeks to allow for fibrosis, which may aid in suture repair [6].

Lastly, the closure must be considered for the wound to heal ultimately; but primary closure is not always possible. In these cases, free flaps can be beneficial due to increased blood flow to the wounded area and improved healing secondary to increased antimicrobial properties and nutritional resources [8,10]. Muscle flaps may also be considered but are less advantageous as they require skin grafting [8,10].

## Conclusions

Automatic washing machines are more commonly associated with minor extremity injuries in the pediatric population; however, injuries can be limb and life-threatening in all patient populations. After ruling out life-threatening injuries, attention should be turned to the perfusion of the limb followed by the assessment and repair of bones, tendons, nerves, and skin. This case describes a severe upper extremity injury that utilized complex reconstructive strategies to salvage a limb successfully with minimal loss of function.
